# Prognostic value of inhibitors of apoptosis proteins (IAPs) and caspases in prostate cancer: caspase-3 forms and XIAP predict biochemical progression after radical prostatectomy

**DOI:** 10.1186/s12885-015-1839-z

**Published:** 2015-10-27

**Authors:** Gonzalo Rodríguez-Berriguete, Norelia Torrealba, Miguel Angel Ortega, Pilar Martínez-Onsurbe, Gabriel Olmedilla, Ricardo Paniagua, Manuel Guil-Cid, Benito Fraile, Mar Royuela

**Affiliations:** 1 Department of Biomedicine and Biotechnology, University of Alcalá, 28871 Alcalá de Henares, Madrid Spain; 2 Department of Pathology, Príncipe de Asturias Hospital, 28805 Alcalá de Henares, Madrid Spain; 3 Department of Urology, Príncipe de Asturias Hospital, 28805 Alcalá de Henares, Madrid Spain

**Keywords:** Apoptosis, Caspases, Biochemical progression, Inhibitors of apoptosis proteins, Prostate cancer

## Abstract

**Background:**

The expression status of apoptotic regulators, such as caspases and inhibitors of apoptosis proteins (IAPs), could reflect the aggressiveness of tumors and, therefore, could be useful as prognostic markers. We explored the associations between tumor expression of caspases and IAPs and clinicopathological features of prostate cancer – clinical and pathological T stage, Gleason score, preoperative serum PSA levels, perineural invasion, lymph node involvement, surgical margin status and overall survival – and evaluated its capability to predict biochemical progression after radical prostatectomy.

**Methods:**

Protein expression of caspases (procaspase-8, cleaved caspase-8, procaspase-3, cleaved caspase-3, caspase-7 and procaspase-9) and IAPs (cIAP1/2, cIAP2, NAIP, Survivin and XIAP) was analyzed by immunohistochemistry in radical prostatectomy samples from 84 prostate cancer patients. Spearman’s test, Kaplan-Meier curves, and univariate and multivariate Cox proportional hazard regression analysis were performed.

**Results:**

cIAP1/2, cIAP2, Survivin, procaspase-8, cleaved caspase-8, procaspase-3 and caspase-7 expression correlated with at least one clinicopathological feature of the disease. Patients negative for XIAP, procaspase-3 or cleaved caspase-3 had a significantly worse prognosis. Of note, XIAP, procaspase-3 and cleaved caspase-3 were predictors of biochemical progression independent of Gleason score and pathological T stage.

**Conclusions:**

Our results indicate that alterations in the expression of IAPs and caspases contribute to the malignant behavior of prostate tumors and suggest that tumor expression of XIAP, procaspase-3 and cleaved caspase-3 may help to identify prostate cancer patients at risk of progression.

## Background

There are several well-established markers that predict prostate cancer progression after radical prostatectomy, including Gleason grade, pathological stage and preoperative serum PSA [1]. Identification of biological factors that better reflect aggressiveness of tumors could help to improve the prediction capability of the existing makers.

Apoptosis is a type of programmed cell death that ensures the elimination of unnecessary or potentially harmful cells. Caspases constitute a family of cysteine proteases involved in the initiation and execution of the apoptotic program. The apoptotic cascades entail the activation by proteolysis of initiator caspases (caspase-2, −8, −9 and −10), which in turn proteolyze and activate executioner caspases (caspase-3, −6 and −7) [2]. There are two major apoptotic pathways: the extrinsic and the intrinsic (or mitochondrial) apoptotic pathways. These apoptotic pathways converge in the activation of executioner caspases, which proteolyze a plethora of substrates ultimately leading to the death of the cell [3].

Evasion of apoptosis, a characteristic of tumor cells, occurs by alteration in the levels and functions of apoptosis regulators [4]. In this regard, loss of expression of caspases is frequent in several human malignancies, including prostate cancer [5], and has been linked in some cases to poor prognosis [6, 7] and resistance to cell death induced by death receptors and chemotherapeutic compounds [8, 9]. Other important apoptosis regulators frequently altered in human cancers are the inhibitors of apoptosis proteins (IAPs). The IAP family in humans comprises eight members: NAIP (neuronal apoptosis inhibitory protein), XIAP (X-linked inhibitor of apoptosis protein), ILP2 (IAP-like protein 2), cIAP1 (cellular IAP 1), cIAP2, BRUCE (Baculoviral IAP repeat containing ubiquitin-conjugating enzyme), Survivin and Livin (ML-IAP), characterized by containing at least one baculoviral IAP repeat (BIR) domain [10]. IAPs are able to inhibit apoptosis induced by a variety of stimuli through different mechanisms, including direct inhibition of caspases (XIAP), sequestration of pro-apoptotic molecules such as SMAC/DIABLO (cIAP1/2, Survivin, Livin), ubiquitin-mediated degradation and non-degradative inactivation of caspases (cIAP1/2, XIAP), and activation of the pro-survival NF-κB pathway (cIAP1/2, XIAP), among others [10]. In addition, some IAPs can regulate other processes involved in cancer, such as cell cycle, cancer-related inflammation, cell invasion and metastasis [10, 11]. The expression of IAPs has been studied in several types of cancer, such as esophageal [12], colon [13], cervical [14] or prostate [15] cancer.

The aim of the present work was to evaluate the prognostic capability of the tumor expression of a broad panel of IAPs and caspases for biochemical progression after radical prostatectomy, as well as to assess its association with the clinicopathological features of prostate cancer.

## Methods

### Patients

All the procedures were examined and approved by the University of Alcalá and Principe de Asturias Hospital Ethics Committees (PI13/1801; 2013/003/20130214) and were in accordance with the ethical standards of the Committee for Human Experimentation, with the Helsinki Declaration of 1975 (revised in Tokyo 2004) and the Committee on Publication Ethics guidelines. This study was performed with the written consent of the patients or their relatives. All pathological, clinical or personal data were anonymized and separated from any personal identifiers. The present study included 84 men who were diagnosed with prostate cancer and underwent radical prostatectomy as definitive therapy between 1992 and 1999, without receiving pre-surgical treatment, or post-surgical therapy before biochemical progression. Only 40.5 % (*n* = 34) of patients had biochemical progression (32 patient at 5 years and 2 patients between 5 and 10 years). In all patients they were studied lymph node but only six patients are positives. 41.7 % of patients had positive surgical margins. Prostate cancer was detected by serum PSA screening and rectal examination, and diagnostic was confirmed by histopathological examination of needle biopsy cores. The median age (range) at the time of surgery was 66 (52–74). Patients were generally scheduled to have a serum PSA measure every 3 months for the first year and every 6 months thereafter. Patients with PSA persistence after radical prostatectomy were included in the study. Median follow-up (range) time of the cohort was 76.2 (15.6–158.4) months, being defined as the time between the surgery and the biochemical progression or the last record. Clinicopathological features of the patients are shown in Table 1.

**Table 1:** Clinicopathological features of patients

	Median (range)
Age	66 (52–74)
Preoperative serum PSA (ng/ml)	10.3 (0.2-118.0)
	% (n)
Preoperative serum PSA	
<10 ng/ml	41.7 (35)
≥10 ng/ml	58.3 (49)
Gleason score	
<7	28.6 (24)
7	47.6 (40)
>7	23.8 (20)
Clinical T stage	
I	56 (47)
II	44 (37)
Pathological T stage	
II	64.3 (54)
III	32.1 (27)
IV	3.6 (3)
Node involvement	7.1 (6)
Positive surgical margins	41.7 (35)
Perineural invasion	17.9 (15)
Total Biochemical progression	40.5 (34)
at 5 years	38.1 (32)
5 - 10 years	2.4 (2)
Survival	64.7^a^(22)
Deaths	35.3^a^(12)

^a^Expressed respect total biochemical progressión

### Reagents

Total serum PSA was measured by the AxSYM system (Abbott, IL). The following antibodies were from Santa Cruz Biotechnology (Santa Cruz, CA): mouse antihuman caspase-8 (for detection of procaspase-8, used at a 1:25 dilution), caspase-8/p20 (cleaved caspase-8, 1:50), caspase-3 (procaspase-3, 1:25), caspase-3/p20 (cleaved caspase-3, 1:100), caspase-7/p20 (caspase-7, 1:25), caspase-9 (procaspase-9, 1:50) and Survivin (1:75); rabbit antihuman cIAP2 (1:75) and XIAP (1:100); and goat antihuman cIAP1/2 (1:150) and NAIP (1:100). Biotin-conjugated antibodies were from Dako (Barcelona, Spain). Avidin-biotin peroxidase complex (ABC kit) was from Vector Laboratories (Burlingame, CA).

### Immunohistochemical analysis

Immunohistochemistry was performed following the avidin-biotin-peroxidase complex (ABC) method as previously described [15]. Specificity controls for immunohistochemistry were as reported previously [5, 15]. Briefly, for negative controls tissues were incubated with blocking peptides or pre-immune serum (Santa Cruz Biotechnology). Additionally, in five of the samples, one part of the prostate tissues was frozen in liquid nitrogen immediately after surgery and maintained at −80 °C, to be later used for Western blotting analysis in order to test antibody specificity. In this portion, cryostat sections were stained with toluidine blue to confirm the histopathological diagnosis.

Immunostaining in the cancerous epithelium was evaluated by two independent pathologists (P.M.-O. and G.O.), blinded for the outcome measure, in five randomly selected fields per section and six sections per patient. Patients were stratified as positive (those showing staining in more than 5 % of the cancerous epithelium) or negative, as has been previously described [5, 15].

### Statistical analysis

The main outcome measure of the study was time to biochemical progression at 10 years, defined as the time between definitive therapy to the first of at least two consecutive elevations in the total serum PSA level above 0.2 ng/ml. Established prognostic variables included in the study were preoperative serum PSA levels, pathological and clinical T stages (2010 AJCC/UICC TNM classification [16]), postoperative Gleason score, perineural invasion, lymph node involvement, surgical margin status and overall survival. The pathologists undertook a regrading of the samples following the 2005 ISUP consensus [17] and the reassigned Gleason scores were used in the analyses. To evaluate the association between clinicopathological and immunohistochemical variables Spearman’s test was performed. Log-rank test and Kaplan-Meier curves were used for survival comparisons. To explore the correlation of the studied immunohistochemical parameters and the established prognostic variables with biochemical progression, univariate and multivariate Cox proportional hazard regression analyses were performed. All statistical analyses were performed using the SPSS 19.0 software (SPSS Inc. Chicago, IL, USA). *P* values < 0.05 were considered as significant.

## Results

### Expression of IAPs and caspases and its correlation with clinicopathological features

**Fig. 1:**
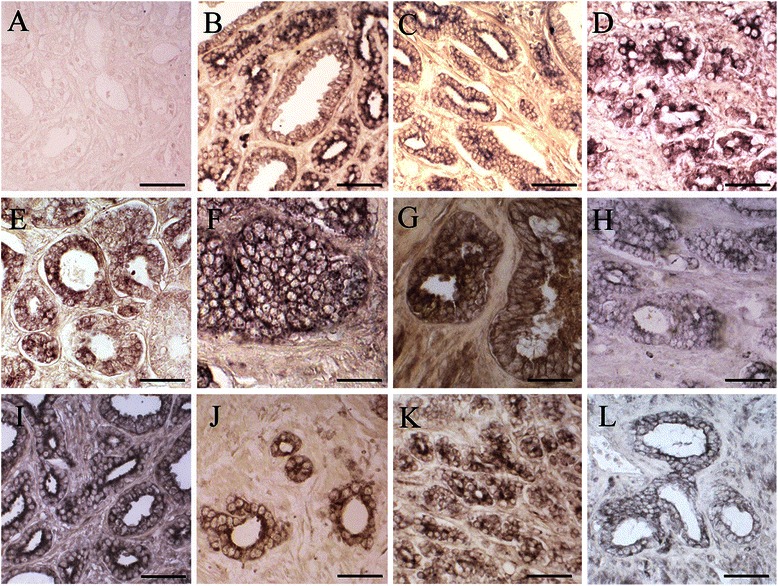
Expression of IAPs and caspases in prostate cancer tissue. As an example, a negative immunoreaction for XIAP (**a**) is shown. Positive tumor immunostaining for cIAP1/2 (**b**), cIAP2 (**c**), NAIP (**d**), Survivin (**e**), XIAP (**f**), procaspase-8 (**g**), cleaved caspase-8 (**h**), procaspase-3 (**i**), cleaved caspase-3 (**j**), procaspase-9 (**k**) and caspase-7 (**l**) is shown. Scale bars: 20 μm (**d**, **f**, **g**, **h**, **j**), 25 μm (**a**, **b**, **e**, **i**, **l**) and 30 μm (**c**, **k**)

Immunohistochemical analysis revealed a predominantly cytoplasmic expression pattern for all the studied proteins (Fig. 1). This part has been previously published [5, 15]. Table 2 shows the percentage (number) of patients with positive immunoreactions for the studied proteins. Spearman’s test evidenced significant positive correlations between a) cIAP1/2 and pathological T stage; b) cIAP2 and positive surgical margins; c) Survivin and perineural invasion; d) procaspase-8 and both clinical and pathological T stages; f) cleaved caspase-8 and preoperative serum PSA; and g) caspase-7 and Gleason score and node involvement (Tables 3 and 4). Moreover, there was a significant inverse correlation between procaspase-3 and positive surgical margins (Tables 3 and 4). Interestingly, expression of XIAP, procaspase-3 and cleaved caspase-3 inversely correlated with the occurrence of biochemical progression, indicating that these proteins can have a role as prognostic markers (Tables 3 and 4). Accordingly, the expression of XIAP and cleaved caspase-3 were positively correlated with overall survival (Tables 3 and 4). On the other hand, Survivin expression was inversely correlated with overall survival.

**Table 2:** Percentage (number) of positive patients for IAPs (left panel) and caspases (right panel)

	% (*n*)		% (*n*)
cIAP1/2	61.90 (52)	Procaspase-8	28.57 (24)
cIAP2	33.33 (28)	Cleaved caspase-8	48.80 (41)
NAIP	58.33 (49)	Procaspase-3	35.71 (30)
Survivin	20.23 (17)	Cleaved caspase-3	57.14 (48)
XIAP	35.71 (30)	Caspase-7	39.28 (33)
		Procaspase-9	38.09 (32)

**Table 3:** Correlation between tumor expression of IAPs and caspases and clinicopahological features

	cIAP1/2		cIAP2		NAIP		Survivin		XIAP	
	r	p	r	p	r	p	r	p	r	p
Preoperative serum PSA	−0.066	0.549	−0.017	0.877	0.069	0.531	0.185	0.091	−0.025	0.228
Pathological T stage	0.219	0.045*	0.019	0.866	0.037	0.735	0.101	0.362	−0.136	0.218
Clinical T stage	0.103	0.349	0.034	0.759	0.118	0.287	0.090	0.414	0.089	0.419
Gleason score	0.153	0.165	−0.124	0.262	0.052	0.641	−0.172	0.118	0.084	0.446
Perineural invasion	0.110	0.320	0.066	0.551	0.079	0.476	0.229	0.036*	−0.088	0.426
Node involvement	0.122	0.267	0.000	1.000	0.047	0.672	−0.025	0.824	0.030	0.455
Positive surgical margins	0.066	0.549	0.222	0.042*	0.127	0.251	0.055	0.619	−0.179	0.109
Biochemical progresion	−0.002	0.983	0.086	0.438	−0.041	0.711	0.188	0.086	−0.210	0.056
Survival	−0.090	0.414	−0.077	0.489	0.119	0.281	0.239	0.019*	−0.331	0.002*

Correlations between immunohistochemical variables (according to the stratification shown in Table 2) and preoperative serum PSA, pathological T stage, clinical T stage and Gleason score were evaluated by Spearman’s test (r, correlation coefficient). *p < 0.05. (Note: significant correlations after Bonferroni correction should be those with a *p* < 0.005)

**Table 4:** Correlation between tumor expression of IAPs and caspases and clinicopahological features

	Procaspase-8		Cleaved caspase-8		Procaspase-3		Cleaved caspase-3		Caspase-7		Procaspase-9	
	r	p	r	p	r	p	r	p	r	p	r	p
Preoperative serum PSA	0.000	1.000	0.294	0.007*	0.076	0.494	−0.098	0.377	0.185	0.091	0.017	0.881
Pathological T stage	0.216	0.048*	−0.056	0.611	−0.154	0.161	0.160	0.146	−0.023	0.833	−0.072	0.513
Clinical T stage	0.235	0.031*	−0.003	0.979	−0.161	0.144	−0.055	0.617	0.121	0.237	−0.054	0.625
Gleason score	0.118	0.287	−0.006	0.954	0.013	0.904	0.107	0.331	0.289	0.008*	−0.151	0.171
Perineural invasion	0.118	0.285	0.104	0.345	−0.023	0.834	0.027	0.808	0.007	0.951	−0.046	0.680
Node involvement	−0.175	0.110	0.007	0.952	−0.110	0.318	0.147	0.183	0.250	0.022*	0.068	0.539
Positive surgical margins	0.160	0.145	−0.149	0.176	−0.277	0.011*	−0.098	0.377	−0.037	0.738	0.133	0.229
Biochemical progresion	0.015	0.890	−0.126	0.254	−0.362	0.001*	−0.364	0.001*	0.082	0.461	0.002	0.983
Survival	0.163	0.139	0.014	0.898	−0.161	0.142	−0.250	0.022*	−0.202	0.065	−0.133	0.229

Correlations between immunohistochemical variables (according to the stratification shown in Table 2) and preoperative serum PSA, pathological T stage, clinical T stage and Gleason score were evaluated by Spearman’s test (r, correlation coefficient). *p < 0.05. (Note: significant correlations after Bonferroni correction should be those with a *p* < 0.005)

### Univariate analysis for time to biochemical progression of tumor expression of IAPs and caspases

**Fig. 2:**
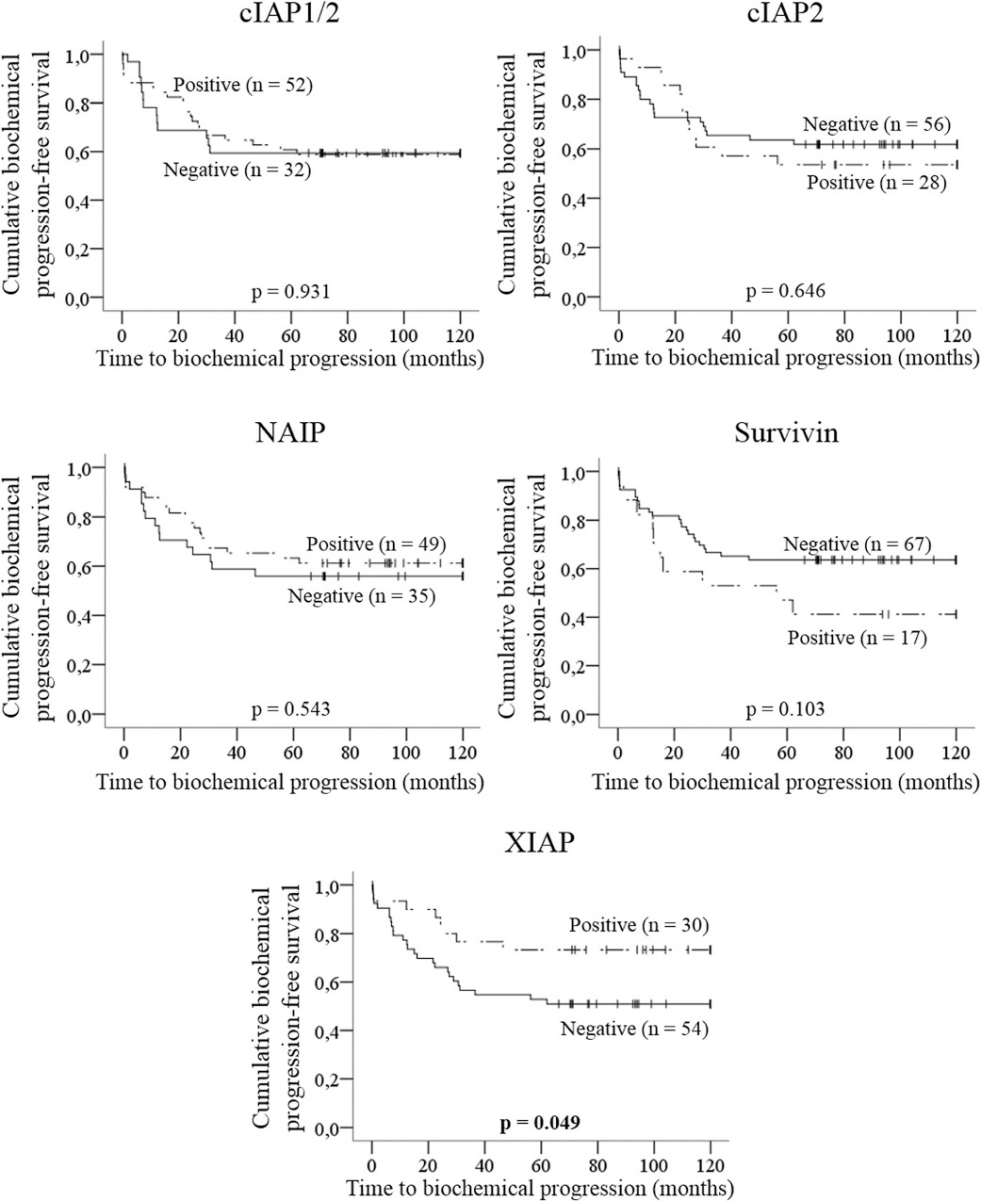
Kaplan-Meier curves for time to biochemical progression according to tumor expression of IAPs. Vertical tick marks represent censored observations. Statistical significance was evaluated by log-rank test (*p* values). Bold value indicates statistical significance

**Fig. 3:**
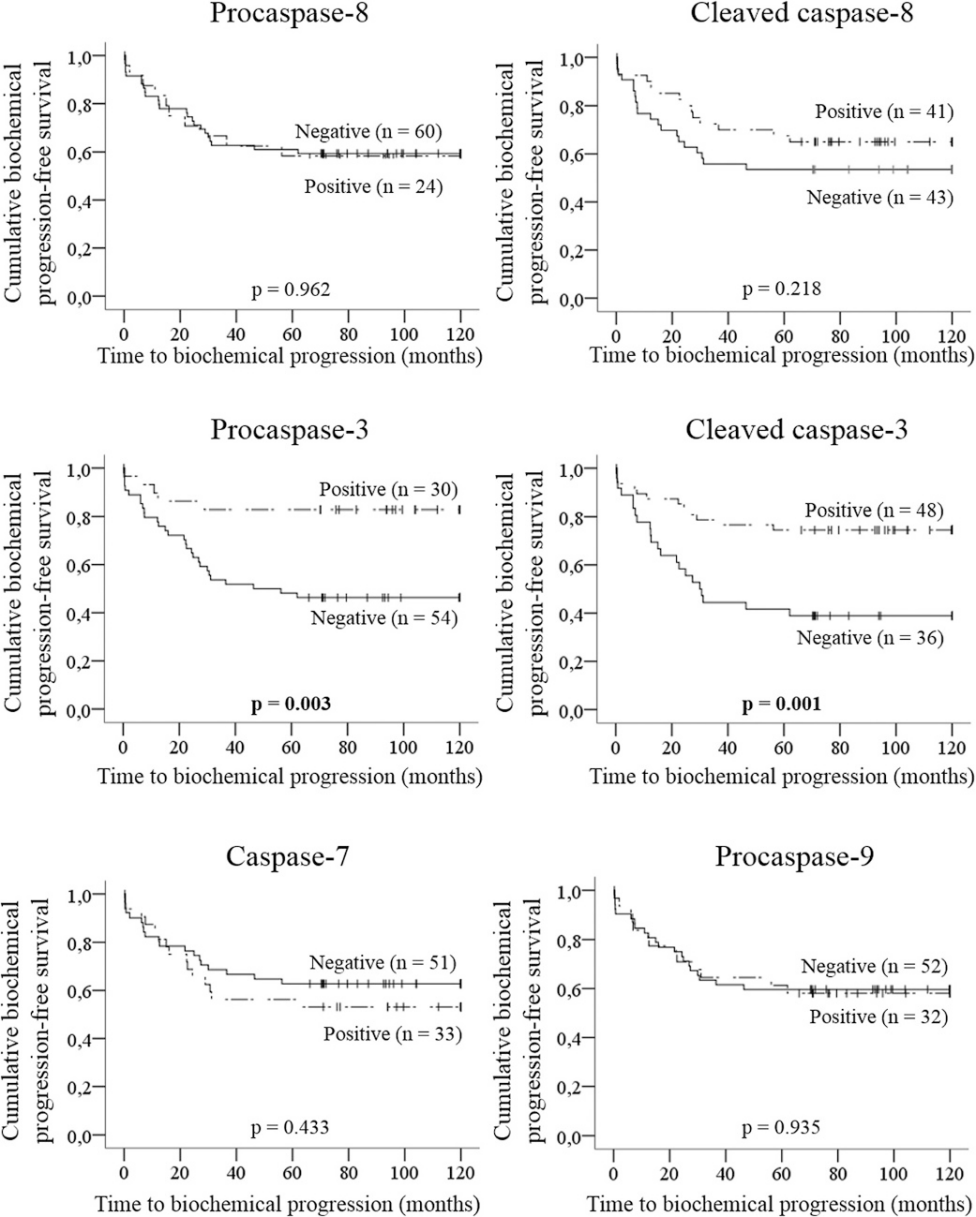
Kaplan-Meier curves for time to biochemical progression according to tumor expression of caspases. Vertical tick marks represent censored observations. Statistical significance was evaluated by log-rank test (*p* values). Bold values indicate statistical significance

Differences between the biochemical progression free-survival times of the groups of patients stratified according to tumor expression of IAPs and caspases were analyzed by the Kaplan-Meier method, using the log-rank as test for significance. Patients negative for XIAP (Fig. 2) and, more markedly, for procaspase-3 and cleaved caspase-3 (Fig. 3) had significantly shorter times to biochemical progression than positive patients.

Univariate Cox proportional hazard regression analysis confirmed the results obtained in the Kaplan-Meier curves (Table 5). Of note, patients negative for procaspase-3, cleaved caspase-3 and, at limit of significance, for XIAP had a higher risk of progression than positive patients (Table 5).

**Table 5:** Univariate Cox proportional hazard regression analysis for time to biochemical progression according to tumor expression of IAPs and caspases

	*p*	HR (95 % CI)		*p*	HR (95 % CI)
cIAP1/2	0.931	0.970 (0.485-1.938)	Procaspase-8	0.962	1.018 (0.487-2.129)
cIAP2	0.646	1.176 (0.588-2.351)	Cleaved caspase-8	0.222	0.653 (0.330-1.294)
NAIP	0.543	0.811 (0.412-1.596)	Procaspase-3	0.005*	**0.260 (0.100-0.672)**
Survivin	0.108	1.831 (0.875-3.834)	Cleaved caspase-3	0.002*	**0.327 (0.161-0.662)**
XIAP	0.055	0.460 (0.208-1.016)	Caspase-7	0.435	1.310 (0.665-2.579)
			Procaspase-9	0.936	1.029 (0.515-2.055)

*Abbreviations: CI* confidence interval, *HR* hazard ratio. Bold values indicate statistical significance. *p < 0.05

### Multivariate Cox proportional hazard regression analysis for time to biochemical progression of XIAP, procaspase-3, cleaved caspase-3 and classic markers

Finally, we assessed the prognostic capability of the immunohistochemical parameters in conjunction with classic markers. In our series, Gleason score, pathological T stage and node involvement, but not preoperative PSA, perineural invasion and positive surgical margins had a prognostic value for biochemical progression in univariate analysis (Table 6). Gleason score and pathological T stage are two of the most important stablished prognostic factors for prostate cancer [1]. For this reason, and to prevent an overfitting of the model, only these two established prognostic factors were introduced in different multivariate Cox hazard regression models, along with the immunohistochemical parameters which resulted significant/borderline significant in the univariate analyses – i.e. XIAP, procaspase-3 and cleaved caspase-3. In the multivariate analysis, XIAP (Table 7A), procaspase-3 (Table 7B) and cleaved caspase-3 (Table 7C) remained as independent prognostic factors after adjusting for the effects of Gleason score and pathological T stage.

**Table 6:** Univariate Cox proportional hazard regression analysis for time to biochemical progression according to clinicopathological features

	*p*	HR (95 % CI)
Preoperative serum PSA	0.199	1.602 (0.780-3.287)
Pathological T stage	0.006*	**2.155 (1.253-3.706)**
Gleason score	0.016*	**1.816 (1.118-2.949)**
Perineural Invasion	0.259	1.579 (0.715-3.489)
Node involvement	0.000*	**5.053 (2.039-12.518)**
Positive surgical margins	0.374	1.357 (0.692-2.661)

*Abbreviations: CI* confidence interval, *HR* hazard ratio. Bold values indicate statistical significance. *p < 0.05

**Table 7:** Multivariate Cox proportional hazard regression analysis for time to biochemical progression of XIAP, procaspase-3 and cleaved caspase-3

A	*p*	HR (95 % CI)
Gleason score	0.023*	1.781 (1.083-2.928)
Pathological T stage	0.035*	1.805 (1.042-3.128)
XIAP	0.042*	0.436 (0.196-0.971)
B	*p*	HR (95 % CI)
Gleason score	0.020*	1.847 (1.104-3.091)
Pathological T stage	0.176	1.470 (0.842-2.568)
Procaspase-3	0.009*	0.268 (0.100-0.718)
C	*p*	HR (95 % CI)
Gleason score	0.007*	2.093 (1.220-3.592)
Pathological T stage	0.001*	2.557 (1.441-4.537)
Cleaved caspase-3	0.000*	0.213 (0.099-0.458)

*Abbreviations: CI* confidence interval, *HR* hazard ratio. *p < 0.05

## Discussion

In spite of the mounting evidence supporting a predominant pro-tumor role of the IAPs in prostate cancer, in the present study, according to the Spearman’s test, only few associations were found between the expression of these proteins and adverse clinicopathological features. Thus, significant positive correlations were found between cIAP1/2 and pathological T stage, cIAP2 and positive surgical margins, and between Survivin and perineural invasion. Furthermore, we found positive correlations between expression of some caspases and adverse clinicopathological features: procaspase-8 correlated with both clinical and pathological T stages, cleaved caspase-8 correlated with preoperative serum PSA, and caspase-7 correlated with Gleason score and the presence of lymph node metastasis. Although at first glance the associations found for caspases may seem contradictory, some points should be considered. First, the pro-apoptotic activity of caspases mostly relies on their cleaved forms and, therefore, higher levels of procaspases do not necessarily entail enhanced apoptotic signaling. Further, there are recent evidences that caspase-8 could exert a pro-tumor role by inducing cell motility, adhesion and metastasis [18]. In this regard, Finlay et al. [19] demonstrated that caspase-8 is involved in the adhesion-mediated activation of the ERK 1/2 signaling pathway. Using caspase-8-null mouse embryo fibroblasts, Helfer et al. [20] demonstrated that caspase-8 is able to promote cell motility and calpain activity under non apoptotic conditions. Interestingly, it has been shown that the role of caspase-8 on cell motility, adhesion and metastasis is independent of the caspase catalytic activity and has been linked to the phosphorylation of procaspase-8 on tyrosine 380, which prevents its cleavage [20]. In addition to these pro-tumor roles of caspase-8, cells with impaired apoptosis despite displaying continued caspase activation have been shown to secrete mitogenic signals in a manner which is dependent on the catalytic activity of caspases [21]. The persistence of these cells could result in increased proliferation of the neighbor cells and, consequently, promote tumor progression [21]. These less-known functions of caspases could also explain why higher expression levels of some of them have been correlated with a poor prognosis in some cancers [22, 23]. All these pro-tumor activities of caspases should be taken into account in future research.

In the present work, we also found that patients with positive immunostaining for XIAP had a significantly better prognosis compared to negative patients. This result is not surprising, as higher XIAP expression has been reported as a favorable prognostic factor in other cancers [24, 25]. Interestingly, it has been shown that TRAMP (**tr**ansgenic **a**denocarcinoma of the **m**ouse **p**rostate) mice deficient in XIAP tend to have a more aggressive disease [26]. In addition, our results are in agreement with a study by Seligson et al. [27] in which higher XIAP expression was also a favorable prognostic factor for biochemical progression after radical prostatectomy. Of note, in that study, despite taking different methodological approaches – regarding the used antibody, the immunostaining scoring and the stratification criteria – XIAP added prognostic value to Gleason score and tumor extension [27], in accordance with the results derived from our multivariate analysis. Therefore, our results add support to previous findings that XIAP may be useful as a predictor of prostate cancer progression, which strongly warrants validation in large prospective studies.

In addition to observations derived from functional studies, the fact that XIAP expression positively associates with a favorable prognosis in some cancers has led to question the pro-tumor role of this IAP. Some authors have argued that this controversy could lie in the initial studies on the anti-apoptotic role of XIAP, which are based on its overexpression in cell lines and the short-term response to different pro-apoptotic stimuli [28]. Thus, when XIAP is stably overexpressed in cell lines at levels comparable to those of tumor cells, it does not protect from apoptosis induced by commonly used chemotherapeutic agents [28]. It has even been recently demonstrated that XIAP is able to mediate cell death through mitochondrial outer membrane permeabilization upon cell detachment [29] or stimulation with resveratrol [30]. It is likely that XIAP functions as an anti- or pro-apoptotic factor, or that is neutral, depending on the scenario. More studies are needed to elucidate the mechanisms accounting for its possible anti-tumor role in patients and its potential as a therapeutic target.

The existing studies on the predictive value of caspases in prostate cancer have focused on assessing the association between the presence of allelic variants of genes encoding for these proteins and the risk of disease or the response to therapy [31, 32]. In the present work, we found that negative expression of either procaspase-3 or cleaved caspase-3 strongly associated with an earlier biochemical progression. As demonstrated in the multivariate Cox models, the prognostic capability of both caspase-3 forms was independent on established prognostic factors – Gleason score and pathological T stage – indicating that they may help to identify patients at high risk of progression. Among the executioner caspases, caspase-3 is thought to be the most determinant in integrating the pro-apoptotic signals coming from both extrinsic and the intrinsic pathways, and ultimately, in triggering the activation of the apoptotic program [3]. Therefore, it is tentative to speculate from our observations that suppression of caspase-3 expression profoundly enhances the survival capability of prostate cancer cells, thus contributing to prostate cancer progression. Strategies aimed to restore or enhance expression of caspases, such as the use of either demethylating agents [33] or the adenovirus-mediated transfer of inducible caspases [34], may be effective for prostate cancer treatment, particularly in those patients lacking tumor caspase-3 expression.

## Conclusions

In summary, we found that tumor expression of cIAP1/2, cIAP2, Survivin, procaspase-8, cleaved caspase-8, procaspase-3 and caspase-7 expression correlates with clinicopathological features of prostate cancer, indicating that these proteins may constitute markers of local disease. Moreover, negative tumor expression of XIAP, procaspase-3 or cleaved caspase-3 predicted early biochemical progression after radical prostatectomy, both alone and after adjusting for the effects of Gleason score and pathological T stage. This adds significance to a previous study evaluating the prognostic capability of XIAP in prostate cancer and others indicating an anti-tumor role for this IAP. Our findings also support the idea that loss of caspase-3 expression in prostate cancer cells strongly decreases their sensitivity to apoptosis, thus contributing to prostate cancer progression. XIAP, procaspase-3 and cleaved caspase-3 may improve the accuracy of the existing markers to predict biochemical progression after radical prostatectomy. Prospective studies in larger cohorts of patients are needed to confirm their prognostic utility.

## References

[1] Swanson GP, Basler JW (2010). Prognostic factors for failure after prostatectomy. J Cancer.

[2] Li J, Yuan J (2008). Caspases in apoptosis and beyond. Oncogene.

[3] Walsh JG, Cullen SP, Sheridan C, Luthi AU, Gerner C, Martin SJ (2008). Executioner caspase-3 and caspase-7 are functionally distinct proteases. Proc Natl Acad Sci U S A.

[4] Hanahan D, Weinberg RA (2011). Hallmarks of cancer: The next generation. Cell.

[5] Rodriguez-Berriguete G, Galvis L, Fraile B, de Bethencourt FR, Martinez-Onsurbe P, Olmedilla G, Paniagua R, Royuela M (2012). Immunoreactivity to caspase-3, caspase-7, caspase-8, and caspase-9 forms is frequently lost in human prostate tumors. Hum Pathol.

[6] Hsia JY, Chen CY, Chen JT, Hsu CP, Shai SE, Yang SS, Chuang CY, Wang PY, Miaw J (2013). Prognostic significance of caspase-3 expression in primary resected esophageal squamous cell carcinoma. Eur J Surg Oncol.

[7] Pingoud-Meier C, Lang D, Janss AJ, Rorke LB, Phillips PC, Shalaby T, Grotzer MA (2003). Loss of caspase-8 protein expression correlates with unfavorable survival outcome in childhood medulloblastoma. Clin Cancer Res.

[8] Eggert A, Grotzer MA, Zuzak TJ, Wiewrodt BR, Ho R, Ikegaki N, Brodeur GM (2001). Resistance to tumor necrosis factor-related apoptosis-inducing ligand (trail)-induced apoptosis in neuroblastoma cells correlates with a loss of caspase-8 expression. Cancer Res.

[9] Fulda S, Kufer MU, Meyer E, van Valen F, Dockhorn-Dworniczak B, Debatin KM (2001). Sensitization for death receptor- or drug-induced apoptosis by re-expression of caspase-8 through demethylation or gene transfer. Oncogene.

[10] Gyrd-Hansen Mand Meier P (2010). Iaps: From caspase inhibitors to modulators of nf-kappab, inflammation and cancer. Nat Rev Cancer.

[11] Church DN, Talbot DC (2012). Survivin in solid tumors: Rationale for development of inhibitors. Curr Oncol Rep.

[12] Nemoto T, Kitagawa M, Hasegawa M, Ikeda S, Akashi T, Takizawa T, Hirokawa K, Koike M (2004). Expression of IAP family proteins in esophageal cancer. Exp Mol Pathol.

[13] Endo T, Abe S, Seidlar HB, Nagaoka S, Takemura T, Utsuyama M, Kitagawa M, Hirokawa K (2004). Expression of IAP family proteins in colon cancers from patients with different age groups. Cancer Immunol Immunother.

[14] Espinosa M, Cantú D, Herrera N, Lopez CM, De la Garza JG, Maldonado V, Melendez-Zajgla J (2006). Inhibitors of apoptosis proteins in human cervical cancer. BMC Cancer.

[15] Rodriguez-Berriguete G, Fraile B, de Bethencourt FR, Prieto-Folgado A, Bartolome N, Nunez C, Prati B, Martinez-Onsurbe P, Olmedilla G, Paniagua R, Royuela M (2010). Role of iaps in prostate cancer progression: Immunohistochemical study in normal and pathological (benign hyperplastic, prostatic intraepithelial neoplasia and cancer) human prostate. BMC Cancer.

[16] Cheng L, Montironi R, Bostwick DG, Lopez-Beltran A, Berney DM (2012). Staging of prostate cancer. Histopathology.

[17] Epstein JI, Allsbrook WC, Amin MB, Egevad LL (2005). The 2005 international society of urological pathology (isup) consensus conference on gleason grading of prostatic carcinoma. Am J Surg Pathol.

[18] Frisch SM (2008). Caspase-8: Fly or die. Cancer Res.

[19] Finlay D, Howes A, Vuori K (2009). Caspase-8 as a potential mediator of pro-tumorigenic signals. Cell Cycle.

[20] Helfer B, Boswell BC, Finlay D, Cipres A, Vuori K, Bong Kang T, Wallach D, Dorfleutner A, Lahti JM, Flynn DC, Frisch SM (2006). Caspase-8 promotes cell motility and calpain activity under nonapoptotic conditions. Cancer Res.

[21] Bergmann A, Steller H (2010). Apoptosis, stem cells, and tissue regeneration. Sci Signal.

[22] Takata T, Tanaka F, Yamada T, Yanagihara K, Otake Y, Kawano Y, Nakagawa T, Miyahara R, Oyanagi H, Inui K, Wada H (2001). Clinical significance of caspase-3 expression in pathologic-stage i, nonsmall-cell lung cancer. Int J Cancer.

[23] Strater J, Herter I, Merkel G, Hinz U, Weitz J, Moller P (2010). Expression and prognostic significance of apaf-1, caspase-8 and caspase-9 in stage ii/iii colon carcinoma: Caspase-8 and caspase-9 is associated with poor prognosis. Int J Cancer.

[24] Ferreira CG, van der Valk P, Span SW, Ludwig I, Smit EF, Kruyt FA, Pinedo HM, van Tinteren H, Giaccone G (2001). Expression of x-linked inhibitor of apoptosis as a novel prognostic marker in radically resected non-small cell lung cancer patients. Clin Cancer Res.

[25] Aggarwal BB, Ichikawa H (2005). Molecular targets and anticancer potential of indole-3-carbinol and its derivatives. Cell Cycle.

[26] Hwang C, Oetjen KA, Kosoff D, Wojno KJ, Albertelli MA, Dunn RL, Robins DM, Cooney KA, Duckett CS (2008). X-linked inhibitor of apoptosis deficiency in the tramp mouse prostate cancer model. Cell Death Differ.

[27] Seligson DB, Hongo F, Huerta-Yepez S, Mizutani Y, Miki T, Yu H, Horvath S, Chia D, Goodglick L, Bonavida B (2007). Expression of x-linked inhibitor of apoptosis protein is a strong predictor of human prostate cancer recurrence. Clin Cancer Res.

[28] Kashkar H (2010). X-linked inhibitor of apoptosis: a chemoresistance factor or a hollow promise. Clin Cancer Res.

[29] Owens TW, Foster FM, Valentijn A, Gilmore AP, Streuli CH (2010). Role for x-linked inhibitor of apoptosis protein upstream of mitochondrial permeabilization. J Biol Chem.

[30] Gogada R, Prabhu V, Amadori M, Scott R, Hashmi S, Chandra D (2011). Resveratrol induces p53-independent, x-linked inhibitor of apoptosis protein (xiap)-mediated bax protein oligomerization on mitochondria to initiate cytochrome c release and caspase activation. J Biol Chem.

[31] Huang SP, Bao BY, Hour TC, Huang CY, Yu CC, Liu CC, Lee YC, Huang CN, Pao JB, Huang CH (2012). Genetic variants in casp3, bmp5, and irs2 genes may influence survival in prostate cancer patients receiving androgen-deprivation therapy. PLoS One.

[32] Mittal RD, Mittal T, Singh AK, Mandal RK (2012). Association of caspases with an increased prostate cancer risk in north indian population. DNA Cell Biol.

[33] Cangemi R, Mensah A, Albertini V, Jain A, Mello-Grand M, Chiorino G, Catapano CV, Carbone GM (2008). Reduced expression and tumor suppressor function of the ets transcription factor ese-3 in prostate cancer. Oncogene.

[34] Shariat SF, Desai S, Song W, Khan T, Zhao J, Nguyen C, Foster BA, Greenberg N, Spencer DM, Slawin KM (2001). Adenovirus-mediated transfer of inducible caspases: a novel “death switch” gene therapeutic approach to prostate cancer. Cancer Res.

